# Inhibiting cancer cell hallmark features through nuclear export inhibition

**DOI:** 10.1038/sigtrans.2016.10

**Published:** 2016-07-01

**Authors:** Qingxiang Sun, Xueqin Chen, Qiao Zhou, Ezra Burstein, Shengyong Yang, Da Jia

**Affiliations:** 1 State Key Laboratory of Biotherapy and Collaborative Innovation Center of Biotherapy, Sichuan University, Chengdu, China; 2 Department of Pathology, West China Hospital, Sichuan University, Chengdu, China; 3 Department of Internal Medicine, UT Southwestern Medical Center, Dallas, Texas, USA; 4 Department of Molecular Biology, UT Southwestern Medical Center, Dallas, Texas, USA; 5 West China 2nd University Hospital, Sichuan University, Chengdu, China

## Abstract

Treating cancer through inhibition of nuclear export is one of the best examples of basic research translation into clinical application. Nuclear export factor chromosomal region maintenance 1 (CRM1; Xpo1 and exportin-1) controls cellular localization and function of numerous proteins that are critical for the development of many cancer hallmarks. The diverse actions of CRM1 are likely to explain the broad ranging anti-cancer potency of CRM1 inhibitors observed in pre-clinical studies and/or clinical trials (phase I–III) on both advanced-stage solid and hematological tumors. In this review, we compare and contrast the mechanisms of action of different CRM1 inhibitors, and discuss the potential benefit of unexplored non-covalent CRM1 inhibitors. This emerging field has uncovered that nuclear export inhibition is well poised as an attractive target towards low-toxicity broad-spectrum potent anti-cancer therapy.

## Introduction

Nuclear export, mainly mediated by the nuclear export factor exportin-1 (better known as chromosomal region maintenance 1, CRM1), is an essential function in all eukaryote that transport nuclear export signal (NES) containing cargoes from the nucleus to the cytoplasm.^1^ Upregulation of this process is a common characteristic for a broad spectrum of cancers; inhibition of nuclear export kills cancer cells effectively, although its anti-cancer mechanism is not conclusive thus far.^2,3^ In addition, CRM1 has been shown to mediate drug resistance.^4,5^ Among dozens of CRM1 inhibitors discovered, a few were clinically tested or are undergoing clinical trials, including the first generation of CRM1 inhibitor, leptomycin B (LMB), and the second-generation CRM1 inhibitor SINE (specific inhibitor of nuclear export).^6^ In this review, we first present the background of nuclear–cytoplasmic transport, the nuclear export factor CRM1 and the cancer hallmark pathways affected by CRM1 inhibition. We then discuss the details of LMB and SINE, with both being covalent CRM1 inhibitors. Finally, we propose non-covalent CRM1 inhibitors as the next generation of anti-cancer drugs, and discuss their advantage over covalent inhibitors.

## Nucleocytoplasmic transport

Eukaryotes are characterized by the presence of the cell nucleus, which is enclosed by a nuclear envelope and separated from the rest of the cell. The nuclear pore complex (NPC) is the sole gateway on the nuclear envelope that governs protein and nucleic acid exchange between the nucleus and cytosol.^7^ Although small molecules are freely permeable across the NPC, permeability is increasingly restricted as the molecular size approaches 30 kDa.^8^ Movement of bigger molecules or more efficient passage of smaller molecules in and out of the nucleus is mediated by active transport of soluble transport factors called karyopherin proteins.^9,10^ The human genome encodes ~20 different karyopherin proteins, functioning as importin (for nuclear import), exportin (for nuclear export) or transportin (both import and export), each being responsible for transporting a set of cargoes (protein or RNA) containing specific sequences/motifs known as nuclear localization signal (NLS) or NES or both.^11–15^ Karyopherin directly binds to exposed NLS or NES, and determine whether the cargo should go to the cytoplasm or nucleus. Diverse mechanisms, such as post-translational modifications (phosphorylation, acetylation, sumoylation, ubiquitination and so on), protein binding masking/unmasking and disease-related NES mutations, regulate cargo’s NES/NLS accessibility and thus its cellular localization.^16–21^

**Figure 1:**
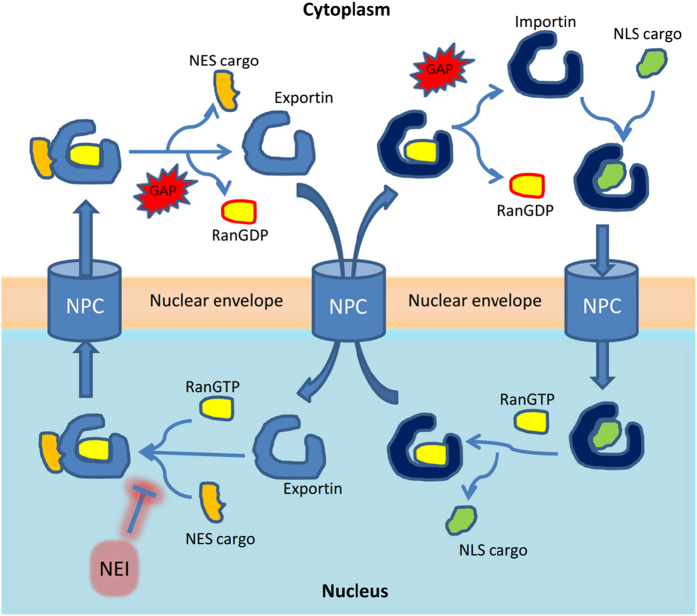
An overview of nucleocytoplasmic transport. Nucleocytoplasmic transport requires cargo with accessible NES or NLS, and its corresponding transport factor exportin or importin. For simplicity, bidirectional keryopherin-mediated transport is omitted. GAP, GTPase-activating protein; NEI, nuclear export inhibitor; NES, nuclear export signal; NLS, nuclear import signal; NPC, nuclear pore complex; RanGDP and RanGTP, GDP- and GTP-bound form of the small GTPase protein Ran.

For nuclear import, a cargo with accessible NLS and an importin form a complex, which is imported into the nucleus together through the NPC (Figure 1).^22,23^ The small GTPase RanGTP in the nucleus then dissociates the cargo from the importin through direct or indirect competition.^24,25^ The RanGTP–importin complex is then recycled to the cytoplasm. After GTP hydrolysis by RanGAP and concomitant RanGDP dissociation, importin is ready for another cycle of nuclear import.^26,27^ For a cargo to exit the nucleus, it must display an NES, which cooperatively forms a tight trimeric complex with an exportin and RanGTP.^28–30^ The complex translocates together into the cytoplasm, where RanGTP is hydrolyzed to RanGDP by RanGAP. This weakens the affinity between NES and exportin, causing dissociation of cargoes.^31^ Bidirectional karyopherins bind to NLS cargoes in the cytoplasm and bind to NES cargoes when exiting nucleus, with similar cargo association/dissociation mechanism to importins and exportins discussed above.^32,33^

## Nuclear export factor CRM1

Of the known exportins, CRM1 is an essential and most often used exportin in cells, which exports numerous cargoes including both proteins and RNAs.^1,34–36^ More than 1050 cargoes have been identified in human cells through proteomic approaches, among which >200 cargoes have been verified through different techniques.^37–40^ CRM1-mediated nuclear export is implicated in various diseases, including cancer, wound healing, inflammation and viral infection. This review will focus on its role in cancer.^6,41,42^ CRM1 is overexpressed in a large variety of tumors including lung cancer,^43^ osteosarcoma,^44^ glioma,^45^ pancreatic cancer,^46^ ovarian cancer,^47,48^ cervical carcinoma,^49^ renal cell carcinoma,^50^ esophageal carcinoma,^51^ gastric carcinoma,^52^ hepatocellular carcinoma,^53^ acute myeloid/lymphoid leukemia,^54,55^ chronic myeloid/lymphoid leukemia,^56^ mantle cell lymphoma,^57,58^ plasma cell leukemia^59^ and multiple myeloma.^59,60^ In addition, CRM1 upregulation is associated with drug resistance and stands out as a poor prognosis factor in many malignancies.^44–46,52,54,61–67^

**Figure 2:**
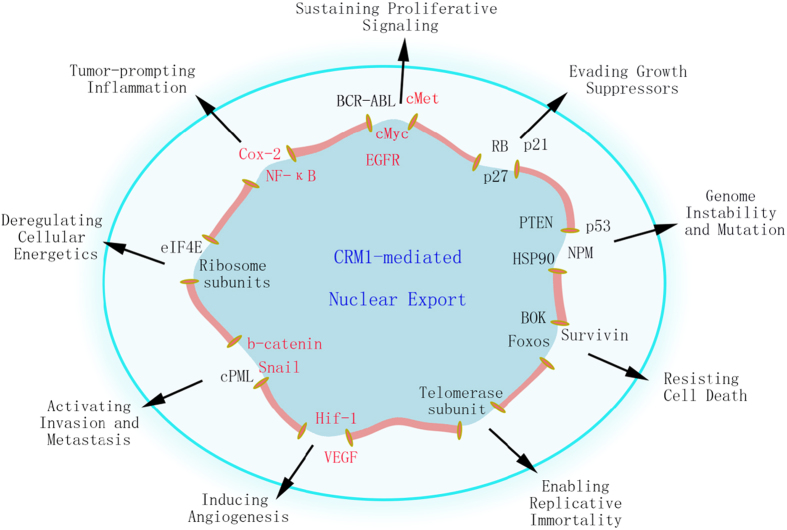
CRM1-mediated nuclear export and cancer hallmarks. CRM1 contributes to the different aspects of cancer hallmarks and CRM1 inhibition may downregulate 9 out of 10 cancer hallmark processes simultaneously. Proteins in black are direct cargoes of CRM1, which are mislocalized to the cytoplasm in various cancer cells. Proteins in red may not be direct cargoes of CRM1, but are shown to be suppressed by nuclear export inhibition through different mechanisms.

CRM1 exports a long list of tumor suppressors or oncogenes, such as p53, FOXOs, p27, nucleophosmin, BCR–ABL, eIF4E and survivin, and these proteins are mislocalized to the cytoplasm in many cancer cell types (Figure 2).^6,68–71^ Furthermore, acting through a variety of mechanisms, CRM1 activates or upregulates the expression of several oncogenic proteins that may not be the direct cargo of CRM1, such as vascular endothelial growth factor, epidermal growth factor receptor, Cox-2, c-Myc and HIF-1 (Figure 2).^63,72,73^ Thus, inhibition of CRM1-dependent nuclear export may affect multiple aspects of carcinogenesis.

## Downregulating cancer hallmarks through CRM1 inhibition

During the multistep development of cancer, cells acquire unique biological properties that enable them to become neoplastic and eventually malignant.^74^ These properties include genomic instability, sustained proliferation, resistance to cell death, reprogramming of cellular energetics and so on, which are summarized as the hallmarks of cancer by Hanahan and Weinberg.^75^ Intriguingly, many CRM1 cargoes are found to be critical for at least nine hallmark features of cancer (Figure 2). Next, we will briefly discuss how the altered cellular distribution of CRM1 cargoes contributes to a particular cancer hallmark, and how CRM1 inhibition may reverse these processes, hopefully bringing some insights into CRM1 inhibitors’ broad-spectrum anti-cancer activity.

### Sustained proliferation

The most remarkable trait that cancer cells acquire is their ability to perpetually divide, resulting in uncontrolled proliferation.^76^ Many tumor-specific mechanisms are involved in this particular cancer cell trait. For instance, the proto oncogene BCR–ABL is formed by a fusion of the ABL1 (Abelson murine leukemia viral oncogene homolog 1) and the BCR (breakpoint cluster region) genes, resulting in a BCR–ABL chimeric protein, which constantly stimulates proliferation of myeloid cells.^77,78^ BCR–ABL is exported to the cytoplasm of cancer cells where it activates the PI3K/Akt pathway.^77,79^ CRM1 inhibition traps BCR–ABL in the nucleus, re-sensitizes leukemia cells to the BCR–ABL inhibitor imatinib, resulting in strong reduction of tumor cell proliferative potential with limited toxicity to normal myeloid precursors.^80,81^ In addition, the expression level of several master growth regulators, such as c-Myc, c-Met and epidermal growth factor receptor, is reduced by CRM1 inhibition through different mechanisms, which might be crucial for the reduced rate of tumor proliferation observed.^53,59,60,82,83^

### Evading growth suppressors

Tumors evade powerful negative regulation of cell proliferation imposed by different growth suppressors such as retinoblastoma protein, p21 and p27.^75^ These cell cycle inhibitors function in the nucleus in normal cells, but are mislocalized to the cytoplasm by CRM1 in various cancers.^84–86^ For example, p27 is a tumor suppressor that functions in the nucleus to inhibit G1 progression in normal cells.^87^ In tumor cells it is rarely mutated, but rather aberrantly exported to the cytoplasm by CRM1, where it is degraded by the proteasome or functions as an oncogene by promoting cell migration.^88–90^ CRM1 inhibition significantly increases nuclear p27 levels and decreases the cytoplasmic oncogenic pool of this protein (Ser10 phosphorylated p27) in tumor cells.^53,90,91^

### Genome instability and mutation

In cancer cells, the DNA maintenance machinery is often mutated or mislocalized, thereby facilitating alterations of the genome and the acquisition of multiple hallmarks subsequently.^75^ p53 is a well-known genome guardian, which has pivotal roles in sensing and repairing DNA damage.^92^ Besides p53 mutations, cancer cells can evade p53 survillience through CRM1-mediated p53 nuclear export.^93,94^ Treatment with CRM1 inhibitors results in increased nuclear p53 level, triggering p53-mediated transcription and apoptosis.^58,95^ Similarly, several other proteins critical for genome stability are exported to the cytoplasm in different types of cancer cells, including HSP90, nucleophosmin and PTEN.^96–98^

### Resisting cell death

In addition to the sustained proliferation ability, cancer cells must bypass programmed cell death by apoptosis.^75^ Survivin, a member of the inhibitor of apoptosis family, is localized in both the nucleus and the cytoplasm of tumor cells, and it is the cytosolic fraction that exerts the cancer-promoting activity.^99,100^ Inhibition of nuclear export by survivin NES antibodies promotes the nuclear accumulation and degradation of survivin, which abolishes its cytoprotective function.^101,102^ In another study, nuclear accumulation of pro-apoptotic protein Bok (Bcl-2-related ovarian killer) by mutation of its NES or CRM1 inhibition causes apoptosis in breast cancer cells.^103^ Another example pertains to FOXO family proteins, which are important transcription factors controlling the expression of apoptosis-related genes.^104^ Through phosphorylation events, their NESs are exposed, leading to FOXO’s cytoplasmic localization and loss of pro-apoptotic activity in cancer cells.^18^

### Enabling replicative immortality

Maintenance of telomeres by telomerase is important for chromosome stabilization and cell immortalization.^105^ As such, telomerase is activated in germ cells and most cancers.^106^ Telomerase RNA subunit TLC1 must be exported into the cytoplasm to recruit the protein subunits for complete assembly of the enzyme, which is then imported into the nucleus to extend telomeres.^107^ Nuclear export of TLC1 requires both CRM1 and the messenger RNA export machinery.^108^ It is reported that nuclear export of TLC1 is an essential step for the formation of the functional RNA containing enzyme, and blocking TLC1 export prevents its cytoplasmic maturation and leads to telomere shortening.^108^

### Inducing angiogenesis

Tumor growth requires new blood vessels formation to supply nutrients for increasing mass of tumor cells.^109^ The well-known prototype of angiogenesis inducer is vascular endothelial growth factor.^110^ CRM1 inhibition causes nuclear retention of the NES-containing cargo Fbw7, a subunit of a ubiquitin ligase that promotes the degradation of nuclear Notch-1 and further leads to decreased vascular endothelial growth factor level.^66^ Copper metabolism MURR1 domain 1 (COMMD1) protein, an inhibitor of HIF-1, is actively exported to the cytoplasm by CRM1 under low oxygen concentrations.^111^ Disruption of the NESs or CRM1 inhibition results in nuclear accumulation of COMMD1, enhancing the repression of transcriptional activity of HIF-1 by COMMD1.^111^

### Activating invasion and metastasis

The transcription factor Snail has important roles in epithelial–mesenchymal transition, tumor invasion and metastasis.^112^ CRM1 inhibition leads to nuclear accumulation of FBXL5 (F-Box and leucine-rich repeat protein 5), which is a negative regulator of Snail.^113^ Silencing CRM1 or Snail results in nuclear accumulation of FBXL5 and inhibition of epithelial–mesenchymal transition.^113^ Similarly, APC (adenomatous polyposis coli) protein, a negative regulator of nuclear β-catenin, is mislocalized to the cytoplasm by CRM1 in cancer cells, resulting in uncontrolled β-catenin transactivation of metastasis-related proteins.^40,114^ Further, cytoplasmic promyelocytic leukemia (cPML) promotes a mesenchymal phenotype and increases the invasiveness of prostate cancer cells through transforming growth factor-β signaling.^115^ cPML nuclear export is mediated by CRM1, co-expression of which with cPML correlates with reduced disease-specific survival in patients.^115^

### Deregulating cellular energetics

Cancer cells usually display upregulated energetic metabolism to adapt to their high rate of proliferation.^75^ The ribosome is an effective cancer drug target because ribosome inhibition limits cellular energetics by affecting global protein synthesis.^116,117^ CRM1-mediated nuclear export is essential for nuclear export of pre-mature ribosome subunits and inhibition of CRM1 causes immature 40S and 60S ribosome production.^118–120^ In addition to ribosome biogenesis, hyperactive translation via eukaryotic translation initiation factor eIF4E is common in the majority of cancers.^121^ eIF4E is abnormally exported to the cytoplasm by CRM1 in cancer cells, together with several proliferative messenger RNAs.^122^ eIF4E cytoplasmic localization in leukemia patients strongly correlates with eIF4E inhibitor treatment outcome.^123^

### Tumor-promoting inflammation

The importance of inflammation in tumor development has been increasingly recognized.^124^ Cox-2 and NF-κB are the key cellular mediators of inflammation that are often upregulated in cancer cells.^125,126^ It is shown that CRM1 inhibitor downregulates Cox-2 level by limiting its messenger RNA export.^72^ Treatment of ovarian cancer cells with a CRM1 inhibitor revealed a reduction in COX-2 expression and concomitant reduction of cell proliferation and increased apoptosis.^47^ NF-κB inhibitor IκBα is also a cargo of CRM1.^127^ IκBα is rapidly locked in the nucleus by CRM1 inhibition and forms a transcriptional inactive complex with NF-κB.^128,129^

Although it is impossible to summarize all proteins involved in nuclear export and cancer, the above examples clearly illustrate the strong link between CRM1 inhibition and reversion of cancer hallmarks. Many mechanism-based cancer drugs only target one particular aspect of cancer. For instance, epidermal growth factor receptor inhibitors reduce cancer proliferation, CDK inhibitors stop cell cycle and BH3 mimetics promote cell death.^130–132^ In response to such single-target therapy, cancer cells may reduce their reliance on a particular protein and develop more dependence on another.^75,133^ Importantly, CRM1-mediated nuclear export is a significant contributing factor in the development of drug resistance.^70,134,135^ As CRM1 inhibition could downregulate 9 out of 10 cancer features simultaneously, it probably would be more effective than targeting a single pathway. Indeed, it has been observed that CRM1 inhibitors have broad-spectrum anti-cancer potency in pre-clinical and clinical studies.

## Different classes of nuclear export inhibitors

**Figure 3:**
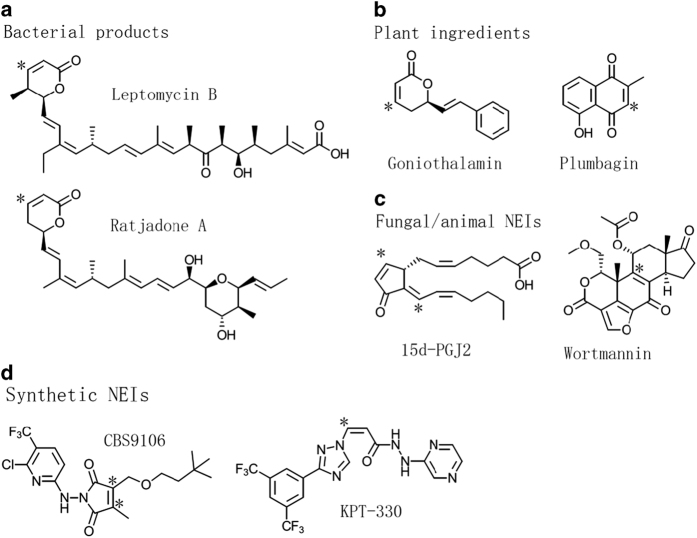
Four classes of nuclear export inhibitors (NEIs). Two representative NEIs from each class is drawn, including (**a**) bacterial products leptomycin B and ratjadone A; (**b**) plant ingredients goniothalamin and plumbagin; (**c**) wortmannin from fungus and 15d-PGJ2 from animals; (**d**) synthetic NEIs CBS9106 and KPT-330. Asterisk (*) denotes possible covalent binding sites to Cys528 on CRM1.

Over the past two decades, plenty of nuclear export inhibitors (NEIs) have been discovered or developed, tested against diseases such as cancer, virus infection and neuronal degeneration.^2,41,134^ By their origins, these inhibitors can be classified into four groups as follows: bacterial products, herbal ingredients, fungal or animal NEIs, and synthetic NEIs (Figure 3). (1) Bacterial NEIs include leptomycin A/B, ratjadone A/C and anguinomycin A/B/C/D, which all have a long polyketide chain with a lactone ring.^136–138^ In general, they are very potent against cancer cells (half-maximal inhibitory concentration below 10 nm), but too toxic and profiling very narrow therapeutic window.^136–138^ (2) Several plant NEIs were discovered from South/Southeast Asia herbs and food additives in recent years, including valtrate, oridonin, acetoxychavicol acetate, curcumin, gonionthalamin, piperlongumine and plumbagin.^62,139–144^ Plant NEIs generally bind/inhibit CRM1 poorly and display mild anti-tumor activity.^62,139–144^ (3) Wortmannin and cyclopentenone prostaglandin (15d-PGJ2) were known for other functions before they were discovered as CRM1 inhibitors.^145,146^ Fungal steroid metabolite wortmannin is a well-known PI3K inhibitor.^147^ The cyclopentenone prostaglandin 15d-PGJ2 is an anti-inflammatory compound produced in the body.^148^ They both have low micromolar NES inhibition potency, which may explain their anti-proliferative and anti-inflammatory properties known earlier.^145,149^ (4) Realizing the significance of NEI in the treatment of cancers and other diseases, scientists have discovered a variety of synthetic inhibitors, including PKF050-638, 5219668, SINEs, compound3/4, CBS9106 and S109.^6,48,145,150–153^ CBS9106 has anti-tumor effect as a single agent in 60 different human cancer cell lines at sub-micromolar concentrations.^154^ SINEs are currently undergoing over 40 clinical trials, for human hematologic malignances and solid tumors (https://clinicaltrial.gov/).

**Figure 4:**
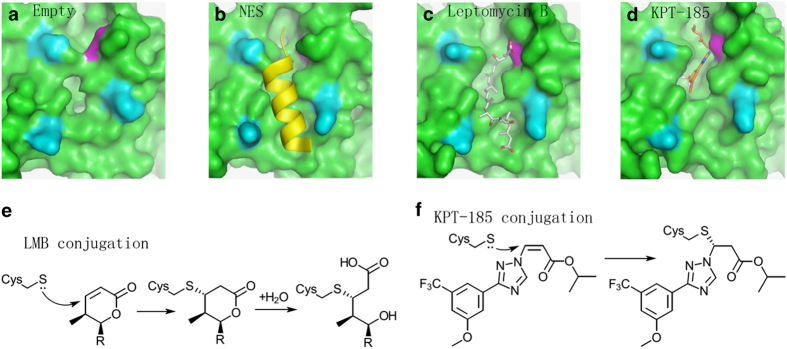
Binding of CRM1 by different molecules. CRM1’s NES-binding grove in the empty (**a**), NES-bound (**b**), leptomycin B-bound (**c**) and KPT-185-bound (**d**) states is shown in green surface representation. The pdb codes are 4HB2, 3GB8, 4HAT and 4GMX respectively. Cys528 is highlighted in purple. NES, LMB and KPT-185 are colored yellow, tint and brown respectively. Three aligned residues are colored cyan to illustrate the orientation of NES groove. (**e**) Mechanism of LMB conjugation. R resembles the long polyketide chain. (**f**) Mechanism of KPT-185 conjugation.

It should be noted that all these inhibitors form a covalent bond with Cys528 of human CRM1 protein, through a Michael addition reaction. Cys528 lies in the NES-binding groove of CRM1 (Figures 4a and b). Thus, these inhibitors directly inhibit NES binding to CRM1 (Figures 4c and d). Mutation of Cys528 disables the anti-tumor action of these compounds.^48,62,70,113,151,152,155^ Next, we will focus on two classes of the most characterized NEIs: LMB and SINEs.

## First-generation NEI: LMB

The first and most well-known inhibitor of CRM1 is LMB.^156^ LMB is a natural product produced by bacteria *Streptomyces*.^157^ It was first identified as an anti-fungal agent.^158^ Later on, it is discovered that LMB potently kills cancer cells.^159^ Scientists had proved that CRM1 is the cellular target of LMB as early as 1998; however, the LMB–CRM1 complex structure was not solved until recently.^160,161^

LMB is a long polyketide molecule with an α,β-unsaturated lactone warhead (Figure 3). In complex with CRM1, its long polyketide chain aligns with the hydrophobic NES-binding groove, forming extensive hydrophobic interactions (Figure 4c). The α,β-unsaturated lactone warhead links to a Cys528 equivalent residue of yeast CRM1 at α-position (Figure 4e). Unexpectedly, the lactone ring of LMB is hydrolyzed, forming an opened up structure of hydroxy acid (Figure 4e). The same hydrolysis is also observed in two other CRM1 inhibitors (anguinomycin A and ratjadone A) from bacteria.^161^ After hydrolysis, LMB forms extra interactions with CRM1, gaining one salt bridge and one hydrogen bond with CRM1.^161^ The hydrolysis is caused by three basic residues (in CRM1) adjacent to the LMB lactone ring, mutation of which results in CRM1 mutant that does not hydrolyze LMB’s lactone ring.^161^

LMB lactone hydrolysis leads to an unexpected finding that the covalent bond between LMB and CRM1 is reversible under certain condition. Using dialysis and pull-down assay, it is shown that LMB with lactone ring de-conjugates from CRM1 (slowly), whereas the hydrolyzed LMB does not.^161^ Thus, hydrolysis prevents de-conjugation of inhibitor and increases inhibitor potency. A permanent CRM1 inhibitor could provide *Streptomyces* bacteria more space to grow by killing fungi more efficiently, as CRM1-mediated nuclear export pathway is essential for fungi.^1^ More importantly, this finding could also explain the clinically strong cytotoxicity of LMB. LMB entered clinical trial for the treatment of cancers in 1996.^136^ However, strong dosage-limiting toxicities produced barely any clinical benefits and the mechanism of toxicity was unknown at the time.^136^ Indeed, recent studies also show that LMB-treated cells have permanently blocked nuclear export, which is lethal not only for cancer cells, but also for normal cells.^1,48,155^

## Second-generation NEI: SINE

Although the early clinical failure of LMB spelled doom for CRM1-targeted drug development, it did not stop the endeavor of academic and pharmaceutical researchers, who discovered NEIs with improved pharmacological properties through library screens, of which PKF050-638 was found as an anti-viral hit.^150^ Its *N*-azolylacrylate scaffold is later on adopted by SINEs.^2^

Like LMB, SINEs also form covalent bond with Cys528 of CRM1. However, SINEs differ from LMB in several aspects (Table 1).^162^ First, SINEs are much more compact when compared with LMB. Whereas LMB occupies almost the entire NES groove, smaller SINEs occupies <50% of the space (Figure 4d). Second, in contrast to LMB, KPT-185 (one of the SINEs) is not hydrolyzed after binding to CRM1 and does not form salt bridge with CRM1 (Figure 4f).^56,163,164^ As such, its covalent bond to CRM1 is found to be slowly reversible.^161^ Third, SINEs but not LMB treatment induces CRM1 degradation, followed by CRM1 re-synthesis after drug removal.^59,165^ Therefore, through bond reversibility and re-synthesis, nuclear export inhibition by SINE treatment is not permanent, but rather transient. It is found that nuclear export in KPT-treated mouse at 10 mg kg^−1^ reversed to ~20% after 1 day and 50% after 3 days.^155^ These observations altogether could explain the fact that SINE is significantly less toxic than LMB observed in clinical trials.

Reported SINEs include KPT-185, KPT-249, KPT-251, KPT-276, KPT-330 and KPT-335. Their half-maximal effective concentration for cancer cells lies between 10 nm to 1 μm, and 5–20 μm for non-neoplastic cells.^133^ KPT-185 is the most studied compound, with limited bioavailability in murine and monkey pharmacokinetic studies.^95^ KPT-276 has been shown to block inflammation and nerve cell damage in mouse models of inflammatory demyelination.^164^ KPT-335 has received a Minor Use/Minor Species designation from the Center for Veterinary Medicine of the Food and Drug Administration for the treatment of lymphomas in canines.

Of all the SINEs, KPT-330 (Selinexor) is the most promising compound and is undergoing numerous human hematologic and solid tumor clinical trials. Before KPT-330’s clinical trial, many expected that inhibiting nuclear export will generate profound side effects, as CRM1 is essential for viability and maintaining the proper localizations of its target cargoes required for normal functions of the cell.^1,166,167^ However, side effects of SINE observed are much milder and controllable, including nausea, vomiting, anorexia, diarrhea, fatigue, weight loss and hepatotoxicity, by no means comparable to chemotherapy treatment.^133,168,169^ The efficacy and toxicity of SINE compounds were reviewed elsewhere recently.^6,63,69,170^

## Future perspective: non-covalent NEIs

To our best knowledge, all known NEIs are covalent CRM1 inhibitors and rely on the conserved Cys528 residue on CRM1 to exert its therapeutic effects. Mutation of Cys528 renders current CRM1 inhibitors inactive.^62,113,155,171,172^ Interestingly, Cys528 mutation has been found in a huge number of fungal CRM1, which provides resistance to the anti-fungal agent LMB.^17^ Here we would like to propose a different class of NEI: non-covalent NEI. Non-covalent NEIs can be used in various cancers like their covalent counterparts by exerting transient inhibition, yet being insensitive to Cys528 mutation. In addition, they may possess intrinsically lower toxicity and higher efficacy.

The majority of drugs in clinical use are non-covalent in nature.^173^ Generally, covalent inhibitors are more prone to non-specific conjugation and causing undesirable off-target effects.^174,175^ All the current NEIs have a Michael acceptor, which is mild reactive and could bind to various targets.^148,161,176^ Side effects of SINE are mainly gastrointestinal and liver related.^168,177,178^ As SINE compounds are taken orally, its higher gastrointestinal concentration may result in more local off-target covalent binding, possibly accounting for the observed side effects.

In addition, SINE resistance can occur in tumor cells expressing very high level of CRM1, and in cells that tumor suppressor proteins are relocalized to cytoplasm after SINE treatment.^155,178,179^ With reduced side effects, non-covalent NEI could be used at higher dosage to achieve tighter transient inhibition, to increase its anti-tumor activity and to improve response rate in patients.

## Conclusion

Cancer is not a single disease, as many different pathways are activated in different cancer cells. Nuclear export factor CRM1 exports/regulates many tumor suppressors and oncoproteins. Notably, CRM1 inhibition can attenuate many cancer hallmarks simultaneously, likely explaining the broad-spectrum anti-cancer potencies observed. The first-generation NEI LMB failed in phase I clinical trial due to high cytotoxicity. Second-generation inhibitors display much reduced cytotoxicity, owing to its reversible inhibition of nuclear export. It should be emphasized that ‘broad-spectrum’ anti-cancer does not imply ‘all-spectrum’, in other words—effective against all cancers. Although KPT-330 clinical trials have been very encouraging, non-covalent NEIs remain an interesting alternative for its possibly low-toxicity and broad-spectrum action as a CRM1-targeted anti-cancer therapy.

## Figures and Tables

**Table 1:** Comparison of LMB and KPT-330’s properties

	*LMB*	*KPT-330*
Covalent binding	Yes	Yes
Hydrolysis reaction	Yes	No
Covalent bond reversibility	No	Yes
CRM1 degradation	No	Yes
CRM1 regeneration	No	Yes
Cellular nuclear export inhibition	Permanent	Transient
Toxicity	High	Low

Abbreviation: LMB, leptomycin B.

LMB results in permanent nuclear export inhibition, whereas KPT-330 induces transient nuclear export inhibition. Although LMB is cinilically highly toxicic, KPT-330 displays much reduced toxicity and is currently undergoing over 40 human clinical trials.
